# LncRNA TAF1A-AS1 regulates the progression in hepatocellular carcinoma by targeting miR-664b-3p/USP22 axis

**DOI:** 10.1007/s12672-026-04454-x

**Published:** 2026-01-26

**Authors:** Huizhao Su, Mei Yao, Yuesong Hao, Zhiqian Wang, Guandou Yuan, Songqing He

**Affiliations:** 1 Division of Hepatobiliary Surgery, The First Affiliated Hospital of Guangxi Medical University, Nanning, 530021 Guangxi China; 2 Guangxi Key Laboratory of Immunology and Metabolism for Liver Diseases, Nanning, 530021 Guangxi China; 3 Key Laboratory of Early Prevention and Treatment for Regional High Frequency Tumor (Guangxi Medical University), Ministry of Education, Nanning, 530021 Guangxi China; 4 Guangxi Key Laboratory of Basic and Clinical Application Research for Hepatobiliary Diseases, Nanning, 530021 Guangxi China

**Keywords:** Hepatocellular carcinoma, TAF1A-AS1, Proliferation, Metastasis, Chemoresistance, MiR-664b-3p, USP22

## Abstract

**Background:**

Long non-coding RNAs (lncRNAs) play an important regulatory role in tumorigenesis and progression. However the role of TAF1A-AS1 in hepatocellular carcinoma (HCC) remains unclear. Therefore, this study aimed to clarify the specific role of TAF1A-AS1 in regulating tumorigenesis and progression of HCC.

**Methods:**

Quantitative real-time PCR (qRT-PCR) was used to determine the expression of TAF1A-AS1, miR-664b-3p and USP22 in tissue samples and cell lines. Functional assays, including Cell Counting Kit-8 (CCK-8) assay, colony formation assay, transwell assays and chemoresistance assay, were performed to study the effects of TAF1A-AS1 in HCC cells. Dual-luciferase reporter assay and RNA immunoprecipitation (RIP) were performed to examine the interaction between TAF1A-AS1 and miR-664b-3p, as well as between miR-664b-3p and USP22. A nude mouse xenograft model was established for the in vivo experiments.

**Results:**

TAF1A-AS1 was remarkably upregulated in HCC tissues, and patients with high TAF1A-AS1 expression had poorer prognosis. Both in vitro and in vivo experiments showed that TAF1A-AS1 promoted the proliferation, invasion and tumorigenicity of HCC cells. Furthermore, mechanism study revealed that TAF1A-AS1 served as a sponge of miR-664b-3p, and USP22 was identified as a downstream target of miR-664b-3p. Additionally, TAF1A-AS1 affected HCC cells sensitivity to sorafenib and activated mTOR signaling through USP22.

**Conclusion:**

Our study demonstrated that TAF1A-AS1 regulated the progression of HCC by sponging miR-664b-3p to activate USP22. These results suggest that TAF1A-AS1 could be a novel HCC prognostic biomarker and a potential therapeutic target.

**Supplementary Information:**

The online version contains supplementary material available at 10.1007/s12672-026-04454-x.

## Introduction

Hepatocellular carcinoma (HCC) is one of the most common malignant tumors and the fourth leading cause of cancer-related deaths in the world [1]. HCC has the characteristics of high malignancy, strong invasion and high recurrence rate, leading to poor prognosis. Despite significant advancements in treatment regimens and medications, the highly heterogeneous nature and stem cell characteristics of HCC contribute to inadequate treatment responses, resulting in a patient survival rate less than 20% over 5 years [2]. This grim statistic underscores the critical necessity for a better understanding of the molecular mechanisms driving HCC progression to facilitate the development of more effective prognostic tools and personalized treatment approaches [3]. Notably, sorafenib is the first-line standard treatment for advanced HCC, but drug resistance remains a major clinical challenge [4]. Therefore, identifying new prognostic markers and therapeutic targets is of great significance to improve the treatment of HCC.

Long noncoding RNAs (lncRNAs) are a type of RNA molecules longer than 200 nucleotides, having regulatory functions without the protein-coding ability [5]. LncRNAs have an important regulatory role in the tumorigenesis, progression and chemoresistance of HCC cells [6]. LncRNAs participate in the pathological and biological activities of HCC, such as cell proliferation, apoptosis, invasion and metastasis [7]. Mechanically, lncRNAs act as molecular sponges for microRNAs (miRNAs) or serve as functional scaffolds recruiting regulatory proteins to their target chromosomal regions [8].

Numerous studies have focused on the functional roles of lncRNAs in cancer. A recent study identified six independent prognostic lncRNAs signatures, including TAF1A-AS1, which was considered to be an independent prognostic biomarker in patients with recurrent colon adenocarcinoma (COAD) [9]. However, the role of TAF1A-AS1 in HCC is unknown. Therefore, in the present study, we aimed at identifying the role of TAF1A-AS1 in regulating HCC tumorigenesis and progression, as well as the molecular mechanisms.

Here, we found that TAF1A-AS1 was involved in the proliferation, invasion, migration, tumor formation and chemoresistance in HCC. Mechanistically, TAF1A-AS1 acted as a molecular sponge of miR-664b-3p and promoted the expression of USP22. Therefore, our finding provided a potential biomarker and therapeutic target for HCC.

## Materials and methods

### Patient samples

Clinical samples were collected from the First Affiliated Hospital of Guangxi Medical University. The use of tissue samples was approved by the Ethics Committee of the First Affiliated Hospital of Guangxi Medical University (No.2025-E0872). All patients provided informed written consent for the purpose of the research.

### Cell lines and cell culture

The HCC cell lines used in the present study were purchased from Cellcook Biotech (Guangzhou, China). These cell lines included LM3, Hep3B, SK-Hep1, Huh7, SNU182, and SNU449. Cells were cultured in RPMI-1640 or DMEM medium at 37 °C under 5% CO_2_/95% air. All media were supplemented with 10% fetal bovine serum and 1% antibiotic/antifungal solution.

### Cell transfection

All plasmids, miRNA mimics, miRNA inhibitors, and their controls used in this article were purchased from Genepharma (Shanghai, China). Cells were seeded on a six-well plate at a density of 5 × 10^5^ cells/well. Transfection operation began after incubation at 37 °C for 24 h in a humidified incubator. All transfections were performed using Lipofectamine™ 3000 (Thermo Scientific, USA) Reagent according to the manufacturer’s instructions.

### Quantitative real-time PCR (qRT-PCR)

We used RNAsimple Total RNA Kit (TIANGEN, China) to extract total RNA from cells and tissues. According to the manufacturer’s instructions, reverse transcription of cDNA was performed using Revert Aid First Strand cDNA Synthesis Kit (Thermo Scientific, USA). qRT-PCR was performed using iTaqTM Universal SYBR^®^ Green Supermix (Bio-Rad, USA). miRNA extraction used miRcute miRNA Isolation Kit (TIANGEN, China); reverse transcription of First-Strand cDNA used miRcute Plus miRNA First-Strand cDNA Kit (TIANGEN, China); qRT-PCR used miRcute Plus miRNA qPCR Kit (TIANGEN, China). Primers used in this study are shown in Table S1.

### Western blot

The cells were homogenized and mixed with RIPA lysis buffer with 1 mM PMSF. The protein concentration was determined using a BCA protein assay kit. Proteins were separated by 10% SDS-PAGE and transferred onto a PVDF membrane. The membrane was incubated in the primary antibody at 4 °C overnight. After incubation for 2 h in the secondary antibody, the reaction bands were visualized and photographed using enhanced chemiluminescence agents. Image analysis system was used to quantify the strength of the bands. The antibodies used in this study are shown in Table S2.

### Cell counting kit-8 (CCK-8) assay

The cells were inoculated in 96-well plates at a density of 1 × 10^3^ cells/well. After incubation in a moist incubator at 37 °C for 0, 24, 48, 72 and 96 h, 10 µL of CCK-8 solution was added to each well, and the cells were further incubated. After 2 h, the medium was removed. An enzyme microplate reader recorded the absorbance value at 450 nm of wavelength.

### Plate colony-forming assay

The cells were seeded in six-well plates at a density of 5 × 10^2^ cells/well and cultured for 2 weeks for colony-forming assay. Cells were washed with PBS twice, fixed with methanol/acetic acid (3:1, v/v), and stained with 0.5% crystal violet. Colonies were counted under a microscope.

### Cell migration and invasion assay

Transwell chambers coated with matrigel were used to detect cell invasion. HCC cells were seeded on the top of the matrix in the upper chamber, and the bottom chamber was filled with medium-containing serum. After 24 h, the cells that migrated into the coating layer of matrigel were fixed with paraformaldehyde and then stained with crystal violet. Cells were observed, counted, and photographed under a microscope. The cell migration assay was similar to the invasion assay, except that the Transwell chamber was not coated with matrigel.

### In vivo assay of tumor growth

Six-week-old male BALB/c nude mice were obtained and reproduced in condition without specific pathogens. SK-Hep1 cells with low expression of TAF1A-AS1 or Huh7 cells overexpressing TAF1A-AS1 or control cells were subcutaneously injected into the mice (6 mice/group). Within 4 weeks, tumor formation in mice was assessed by measuring tumor volume. Then, the tumor was resected and weighed. All animal experiments were conducted in accordance with the ethics guidelines.

### Dual-luciferase reporter assay

The sequences of wild- or mutant-type *TAF1A-AS1* were inserted into PmiRGLO dural-luciferase reporters. Thereafter, the recombinant plasmids and miR-664b-3p mimics or miRNA-NC were cotransfected into 293 T cells by Lipofectamine™ 3000. After transfection for 48 h, the luciferase assay system was used to determine the relative luciferase activity normalizing to renilla luciferase activity. The binding between *USP22* and miR-664b-3p was verified using a similar method.

### RNA immunoprecipitation (RIP) analysis

RIP was performed using Magna RIP RNA Binding Protein Immunoprecipitation Kit according to the manufacturer’s instructions. In short, cell lysates were incubated with magnetic beads conjugated with negative control normal IgG or human anti-Ago2 antibody (Millipore). The immunoprecipitated RNA was then extracted and detected by qRT-PCR to confirm the enrichment of the bound target, and the product was subjected to agarose gel electrophoresis.

### Statistical analysis

All values were expressed as mean ± standard deviation. Data were evaluated using one-way analysis of variance or Student’s t-test. Spearman’s correlation coefficient was used to calculate the correlation between two groups. Kaplan-Meier analysis was used for survival analysis, and the log rank test was used to estimate the difference in survival probability. A P value < 0.05 was considered statistically significant. All data were statistically analyzed using SPSS 21.0 statistical software (SPSS, Inc., USA).

## Results

### Expression of TAF1A-AS1 is upregulated in HCC and correlates with poor prognosis

**Fig. 1:**
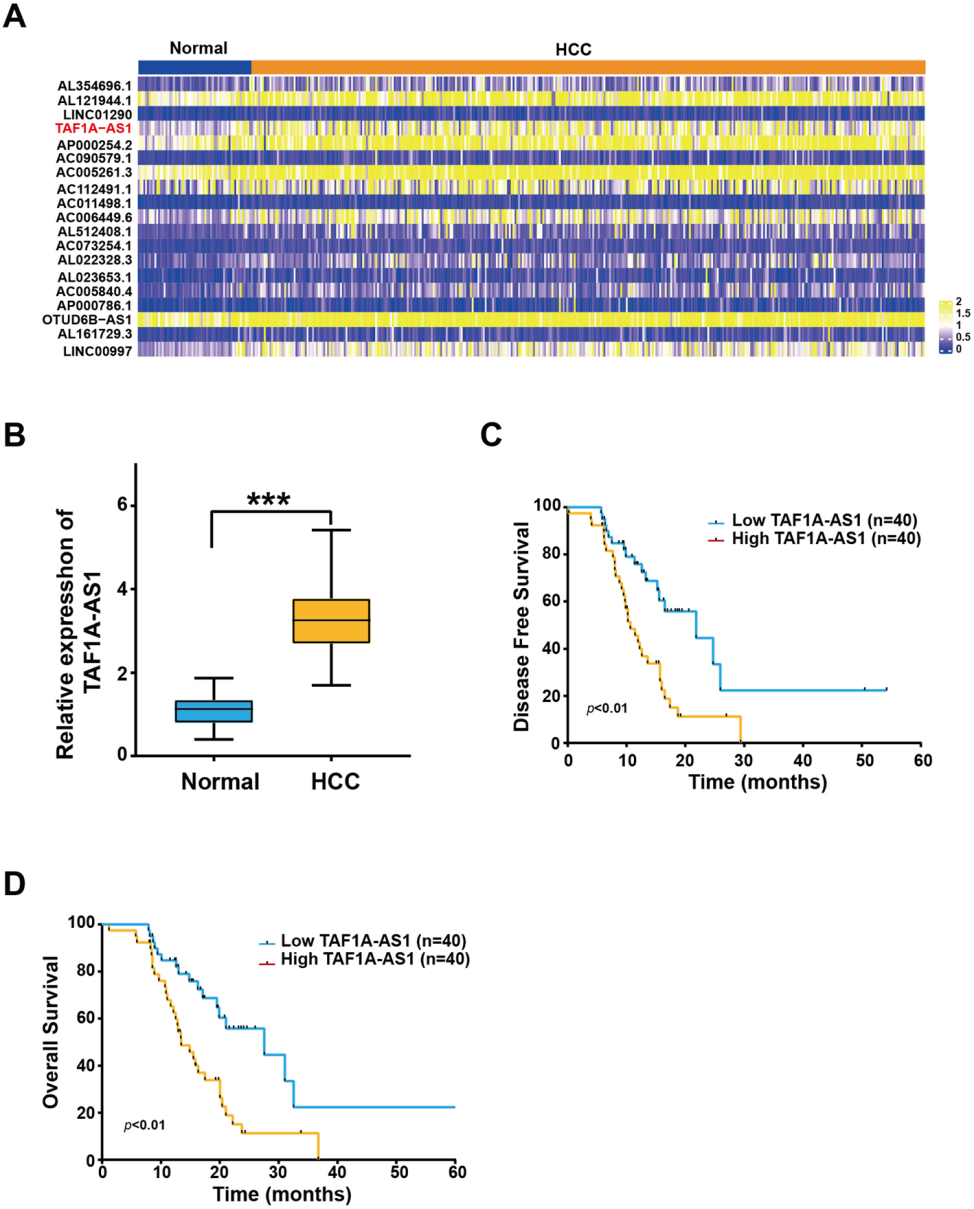
TAF1A-AS1 is upregulated in HCC and correlated with poor prognosis. **A** Heatmap of lncRNAs differentially expressed between adjacent normal liver tissues (50 cases) and hepatocellular carcinoma (HCC) tissues (371 cases) from the TCGA database. **B** The comparative expression of TAF1A-AS1 in 80 pairs of HCC and adjacent normal tissues of tumors by qRT-PCR. **C, D** Kaplan-Meier analysis of the correlation between the TAF1A-AS1 level and HCC prognoses in the 80 patients (**C** disease-free survival; **D** overall survival). ***, *P* < 0.001

We first analyzed The Cancer Genome Atlas (TCGA) database to compare the expression level of TAF1A-AS1 between normal liver tissues (50 cases) and HCC tissues (371 cases). We found that TAF1A-AS1 was significantly upregulated in HCC tissues (Fig. 1A). We detected the expression level of TAF1A-AS1 in our own clinical samples and confirmed that its expression was also higher in HCC tissues than in adjacent normal tissues (Fig. 1B). Kaplan-Meier survival analysis confirmed that higher levels of TAF1A-AS1 expression were significantly associated with poor prognosis both in disease-free survival and overall survival (Fig. 1C and D). These data suggest that TAF1A-AS1 may be an oncogenic lncRNA to promote HCC progression.

### TAF1A-AS1 promotes proliferation and tumorigenicity of HCC cells both in vitro and in vivo

**Fig. 2:**
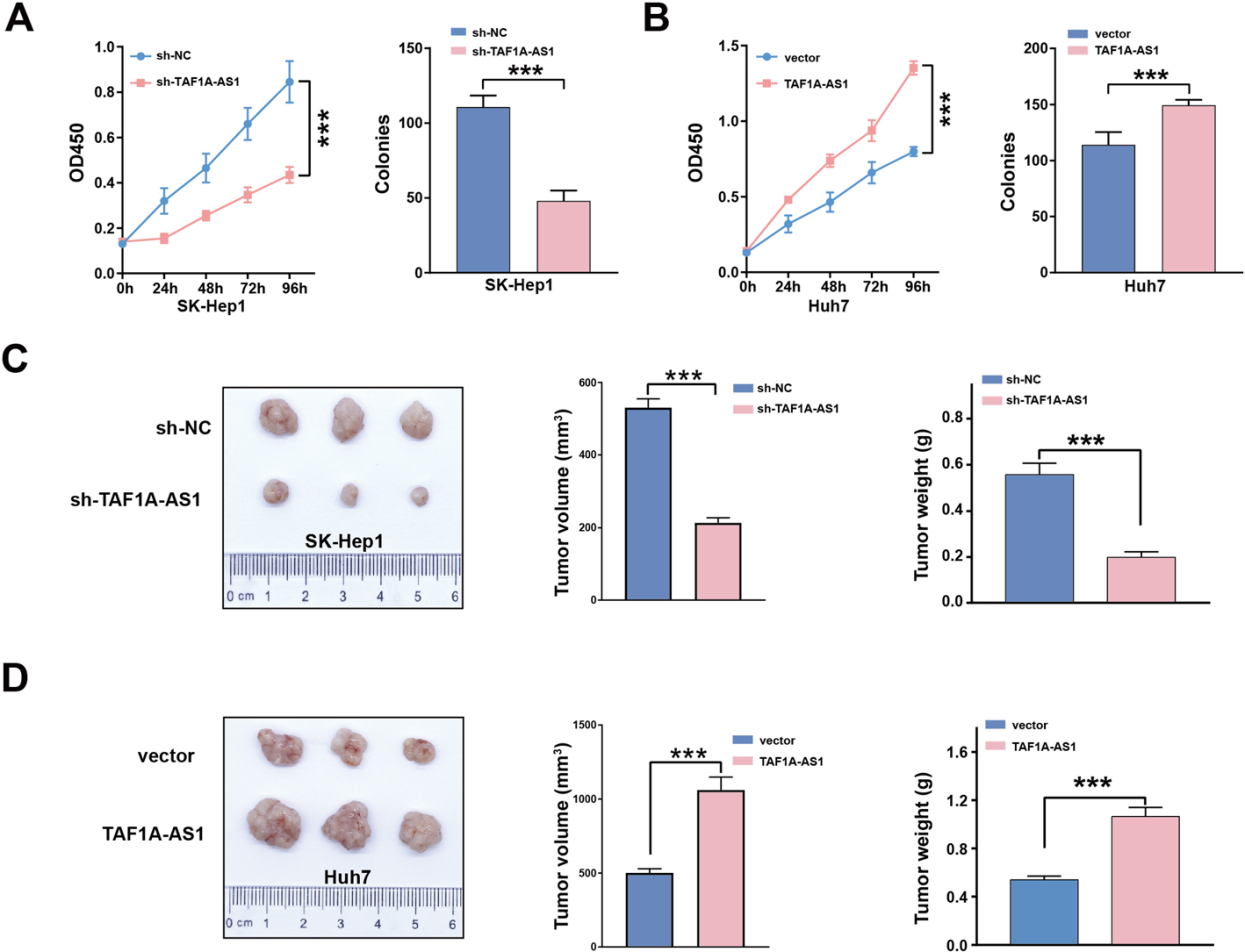
TAF1A-AS1 promotes HCC cell proliferation in vitro and tumor formation in vivo. **A, B** The role of TAF1A-AS1 in cell proliferation was evaluated by CCK8 and colony formation assays. **C** Knockdown of TAF1A-AS1 in SK-Hep1 cells inhibited tumor growth in vivo. **D** Overexpression of TAF1A-AS1 in Huh7 cells promoted tumor growth in vivo. ***, *P* < 0.001

To investigate the potential role of TAF1A-AS1 in HCC progression, we first measured the baseline expression of TAF1A-AS1 in six HCC cell lines (Supplementary Fig. S1A). Based on this result, we selected SK-Hep1 cells for knockdown the TAF1A-AS1 and Huh7 cells for overexpression the TAF1A-AS1. The efficiency of TAF1A-AS1 knockdown and overexpression was detected by qRT-PCR (Supplementary Fig. S1B). Compared with the control group, knockdown of TAF1A-AS1 strongly inhibited proliferation and colony formation in SK-Hep1 cells (Fig. 2A), whereas the overexpression of TAF1A-AS1 promoted proliferation and colony formation in Huh7 cells (Fig. 2B). Then, we confirmed the oncogenic role of TAF1A-AS1 in vivo. The results revealed that knockdown of TAF1A-AS1 significantly decreased the tumor size and weight (Fig. 2C). In contrast, overexpression of TAF1A-AS1 led to increase the tumor size and weight (Fig. 2D).

### TAF1A-AS1 promotes migration, invasion, and epithelial-mesenchymal transition (EMT)-related genes expression of HCC cells

**Fig. 3:**
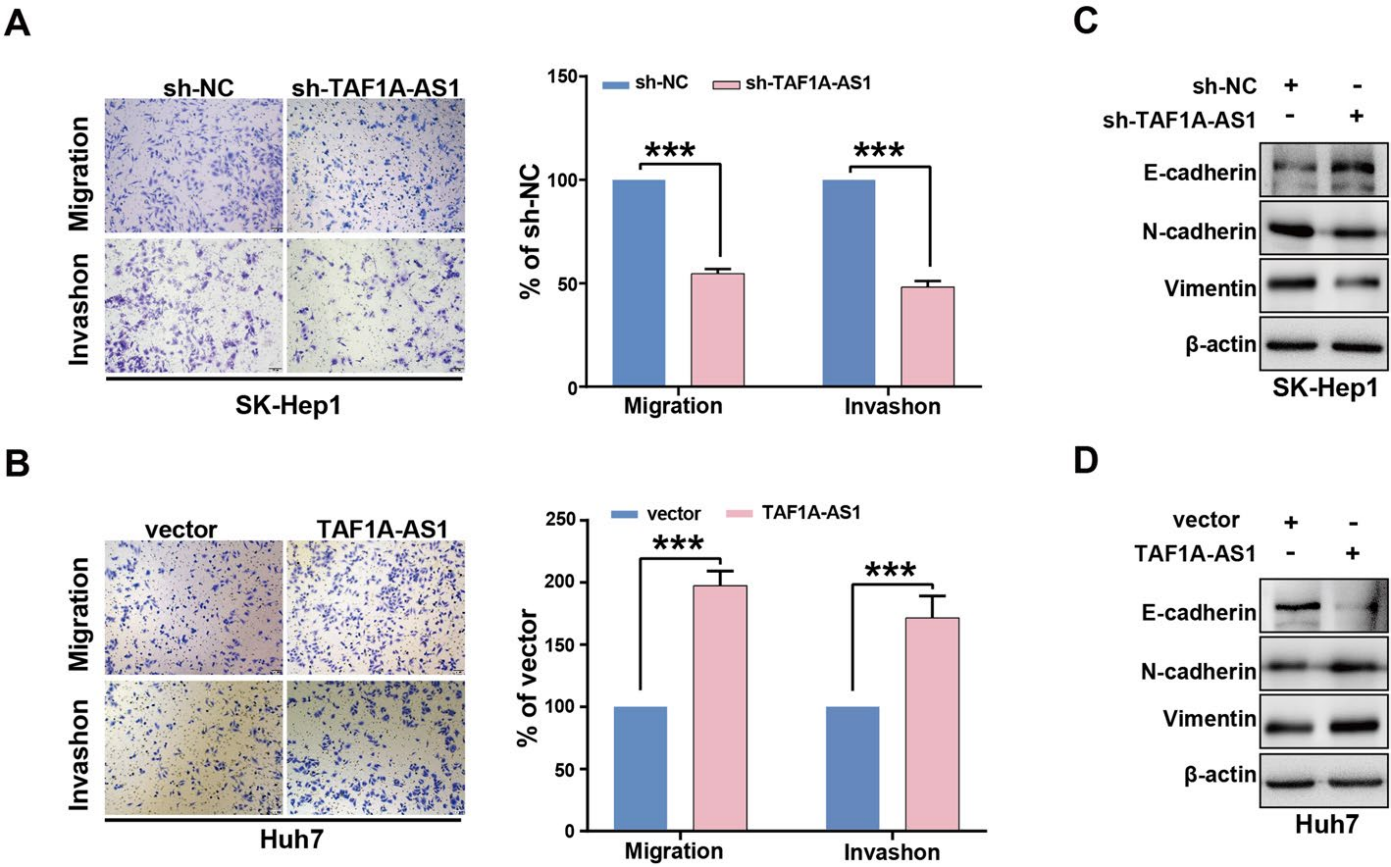
TAF1A-AS1 promotes HCC cell migration, invasion and expression of EMT-related genes in vitro. **A** The migration ability of Sk-Hep1 cells was determined by transwell assay. **B** The invasion ability of Huh7 was determined by transwell assay. **C** The expression of EMT biomarkers was determined by Western blotting in SK-Hep1 cells. **D** The expression of EMT biomarkers was evaluated by Western blotting in Huh7 cells. ***, *P* < 0.001

In addition, we evaluated the effect of TAF1A-AS1 on HCC cells migration and invasion ability using the Transwell assay. Compared with control cells, knockdown of TAF1A-AS1 significantly reduced migratory and invasive abilities in SK-Hep1 cells (Fig. 3A). Conversely, overexpression of TAF1A-AS1 in Huh7 cells led to increase these two malignant capabilities (Fig. 3B). EMT is a key biological process driving cancer cell migration and invasion, then we next examined the expression of EMT-related markers. As shown in Fig. 3C, after suppression of TAF1A-AS1 in SK-Hep1 cells, the expressions of epithelial marker E-cadherin proteins were increased, whereas mesenchymal markers N-cadherin and Vimentin proteins were decreased, but overexpression of TAF1A-AS1 in Huh7 cells exerted the opposite results.

### USP22 is the downstream target of TAF1A-AS1 and mediates TAF1A-AS1 pro-oncogenic effects on HCC cells.

**Fig. 4:**
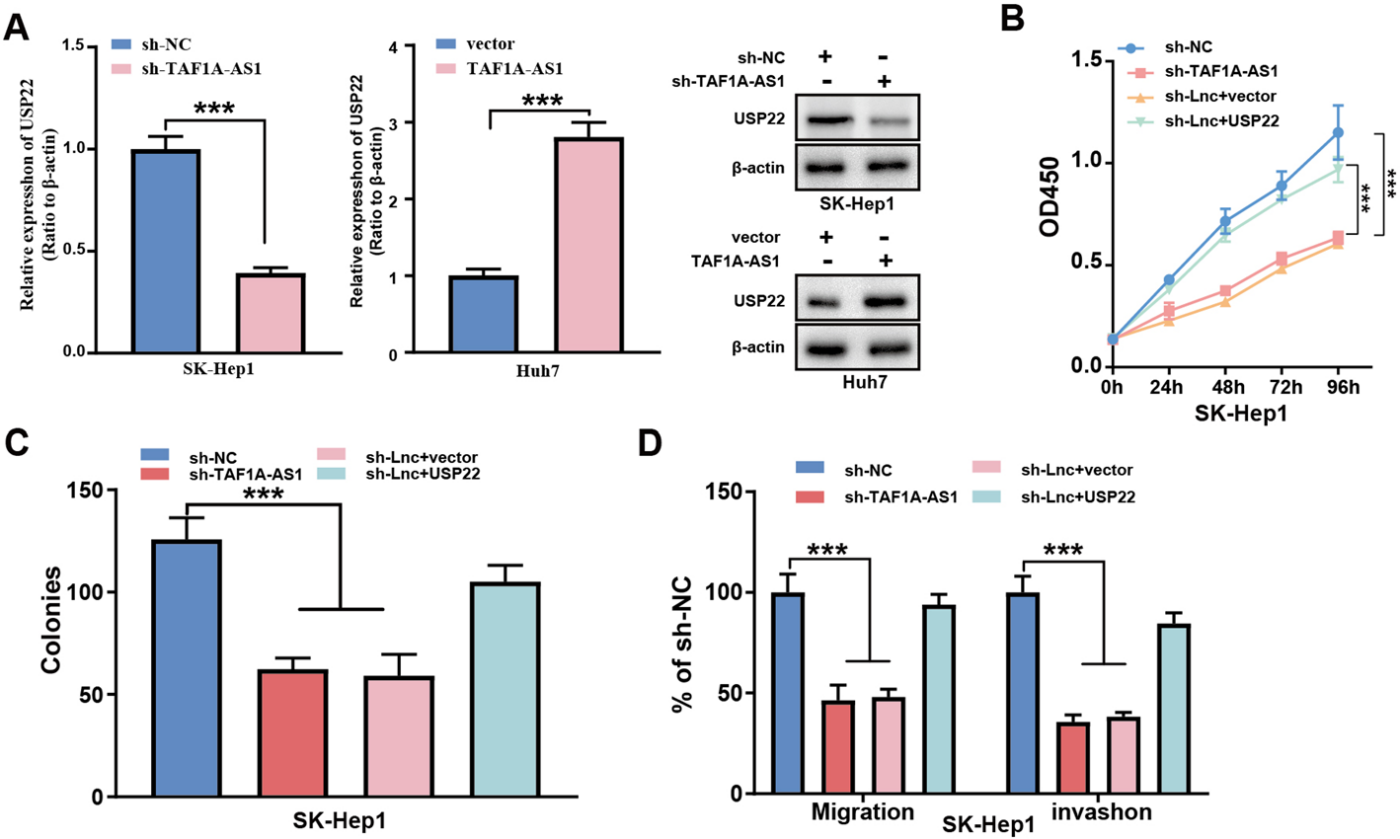
USP22 mediates TAF1A-AS1 pro-oncogenic effects on HCC cells. **A** The expressions of USP22 in SK-Hep1 or Huh7 cells were detected by qRT-PCR and western blot after knockdown or overexpression of TAF1A-AS1, respectively. **B-D** The inhibitory effect on cell proliferation, colony formation, migration and invasion in SK-Hep1 cells by knockdown of TAF1A-AS1 was rescued when USP22 was overexpressed. ***, *P* < 0.001

USP22 has been identified as an oncogene that is overexpressed in a range of malignant tumors, including colorectal cancer and HCC [10, 11]. In the TCGA database, the expression level of USP22 was significantly upregulated in HCC tissues (Supplementary Fig. S2A). Further, analysis of our own clinical samples showed that the expression level of USP22 in HCC samples was also higher in HCC tissues (Supplementary Fig. S2B). To explore whether USP22 is regulated by TAF1A-AS1, we detected USP22 expression after modulating TAF1A-AS1 levels. As shown in Fig. 4A, knockdown of TAF1A-AS1 reduced the expression of USP22 at both mRNA and protein levels, whereas overexpression of TAF1A-AS1 increased the expression of USP22 (Fig. 4A). To confirm whether USP22 mediates pro-oncogenic effects of TAF1A-AS1, we transfected USP22-overexpression vector or control into SK-Hep1 cells with TAF1A-AS1 knockdown. As shown in Fig. 4B-D, overexpressing USP22 rescued the inhibition of proliferation, colony formation, migration and invasion caused by TAF1A-AS1 knockdown.

### TAF1A-AS1 upregulates the expression of USP22 by sponging miR-664b-3p

**Fig. 5:**
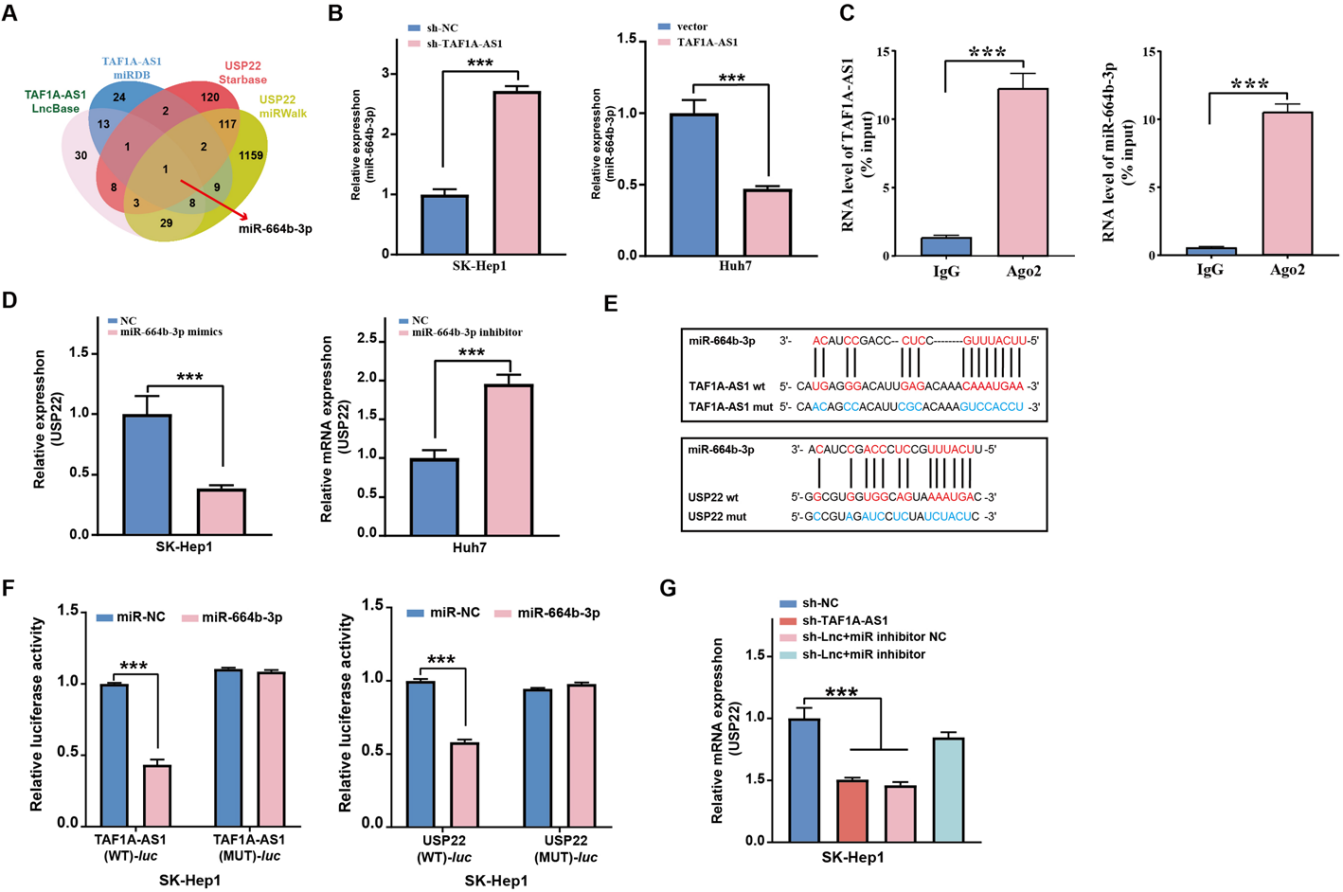
TAF1A-AS1 upregulates the expression of USP22 by sponging miR-664b-3p. **A** Venn diagram showed the predicted target genes of miR-664b-3p. **B** The expression of miR-664b-3p was determined by qRT-PCR after knockdown or overexpression of TAF1A-AS1, respectively. **C** RIP confirmed the interaction between TAF1A-AS1 and miR-664b-3p. **D** The expression of USP22 was evaluated by qRT-PCR after transfecting miR-664b-3p inhibitors or miR-664b-3p mimics, respectively. **E** The binding sites of miR-664b-3p with TAF1A-AS1 and USP22 predicted by LncBase Predicted v.2 and Starbase 3.0 and the indicated mutant constructs were established. **F** The dual-luciferase reporter gene detects the luciferase activity of *TAF1A-AS1* and *USP22* in response to miR-664b-3p mimics or inhibitors. **G** The regulatory effect of TAF1A-AS1 and miR-664b-3p on the expression level of USP22 was determined by qRT-PCR in SK-Hep1 cells. ***, *P* < 0.001

LncRNAs can act as miRNA sponges to prevent them from repressing their target mRNAs. To identify potential miRNAs that interact with both TAF1A-AS1 and USP22, we performed bioinformatics analysis using LncBase, miRDB, Starbase, and miRWalk databases. We finally locked miR-664b-3p as a candidate, as it contains predicted binding sites for TAF1A-AS1 and USP22 (Fig. 5A). Previous studies have reported that miR-664b-3p exerts antitumor effects and represses the proliferation and invasion in HCC cells [12]. Therefore, we selected miR-664b-3p for further investigation. The qRT-PCR analysis showed that the knockdown of TAF1A-AS1 increased the level of miR-664b-3p, whereas TAF1A-AS1 expression decreased the level of miR-664b-3p (Fig. 5B). In order to confirm the direct interaction between TAF1A-AS1 and miR-664b-3p, RIP assay was performed. As shown in Fig. 5C, TAF1A-AS1 and miR-664b-3p were specifically enriched in Ago2-related complexes but not in the IgG control. This result indicated that miR-664b-3p is the authentic target miRNA of TAF1A-AS1.

Further, we explored whether miR-664b-3p regulates USP22. Transfection with miR-664b-3p inhibitor significantly increased the mRNA levels of USP22 in SK-Hep1 cells, whereas overexpression of miR-664b-3p inhibited USP22 expression in Huh7 (Fig. 5D).

Using online databases LncBase Predicted v.2 and Starbase 3.0, we identified the putative binding sequences between miR-664b-3p and TAF1A-AS1, as well as between miR-664b-3p and USP22 (Fig. 5E). Dual-luciferase reporter assays showed that miR-664b-3p mimics significantly reduced the luciferase activity in cells transfected with reporters containing TAF1A-AS1 wt or USP22 wt sequences (Fig. 5F). Finally, we investigated whether TAF1A-AS1 upregulated the expression of USP22 by sponging miR-664b-3p. The qRT-PCR results showed that the knockdown of TAF1A-AS1 suppressed the expression of USP22, but this effect was restored by co-transfection of miR-664b-3p inhibitor (Fig. 5G).

### USP22 mediates TAF1A-AS1 induced HCC chemoresistance

**Fig. 6:**
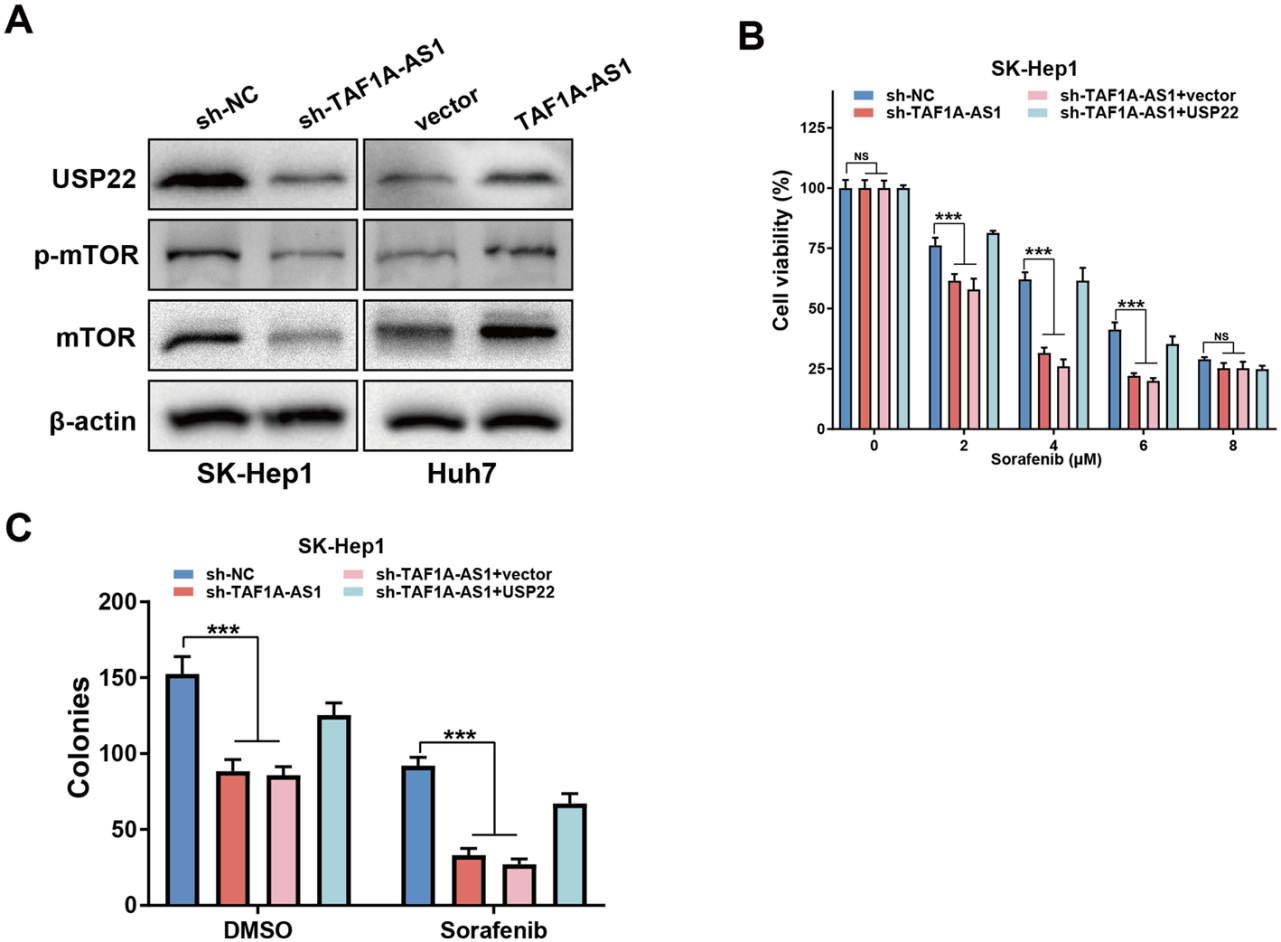
TAF1A-AS1 affects sorafenib sensitivity through USP22/mTOR. **A** Western blot detected the protein levels of USP22, mTOR and p-mTOR after knockdown or overexpression of TAF1A-AS1. **B**, **C** The effect on cell sorafenib sensitivity and colony formation in SK-Hep1 cells by knockdown of TAF1A-AS1 was rescued when USP22 was overexpressed. ***, *P* < 0.001

Recent studies have demonstrated that USP22 can activate the mTOR pathway, thereby promoting tumorigenesis and progression [13–15]. So we wanted to know if TAF1A-AS1 could also activate the mTOR pathway, potentially via USP22. Our results showed that knockdown of TAF1A-AS1 significantly suppressed mTOR and the phosphorylation of mTOR, whereas overexpression of TAF1A-AS1 enhanced mTOR expression and phosphorylation (Fig. 6A). Sorafenib is the first-line standard treatment for advanced HCC, and it inhibits angiogenesis and tumor development by targeting VEGF/VEGFR, Raf/MAPK signaling pathway, and mTOR pathway [16–18]. We further investigated its sensitivity to sorafenib. Our data revealed that knockdown of TAF1A-AS1 significantly strengthened the antiproliferation effect of sorafenib, and this chemosensitization effect was abolished by USP22 overexpression (Fig. 6B, C).

## Discussion

As one of the most common malignant tumors worldwide, HCC is characterized as a highly malignant, recurrent, and drug-resistant cancer. However, we have not yet fully understood the molecular mechanisms of HCC pathogenesis. Recent research evidence shows that lncRNAs play an important role in the regulation of proliferation, invasion, and chemoresistance in HCC [19–23]. Further characterizing and biological functions of HCC-related lncRNAs may enable us to better understand the progression and occurrence of HCC.

To date, the functional role and molecular mechanisms of the lncRNA TAF1A-AS1 in HCC have remained uncharacterized, although one study had reported that it is correlated with the recurrence of COAD [9]. We revealed that the TAF1A-AS1 expression was upregulated in HCC tissues, and it was positively correlated with poor survival prognosis. These results indicated that TAF1A-AS1 might play an important role in HCC progression. In the present study, we systematically explored the potential significance of TAF1A-AS1 involvement in proliferation, migration, invasion and tumorigenesis in HCC cells. Subsequently, our data demonstrated that TAF1A-AS1 enhanced HCC proliferation, invasion and migration. We also found that the knockdown of TAF1A-AS1 repressed the tumor growth of HCC cells in vivo, while overexpression of TAF1A-AS1 promoted tumor growth.

USP22 is highly expressed in a variety of cancers, including HCC [24–26], and USP22 plays a role in promoting the development of cancer by regulating several downstream genes [27–31]. Based on this, we hypothesized that USP22 may be a downstream regulatory target of TAF1A-AS1. The results showed that TAF1A-AS1 positively regulated USP22 expression, and USP22 overexpression reversed the impaired proliferation, invasion and migration caused by TAF1A-AS1 knockdown. These results confirmed that TAF1A-AS1 affected the proliferation, invasion and migration of HCC cells by regulating the expression of USP22.

Based on the above results, we searched for the potential molecular mechanism by which TAF1A-AS1 regulates USP22 expression and subsequently affects HCC progression. Many studies have shown that lncRNAs can interact with miRNA by acting as a ceRNA to prevent miRNA from binding to and inhibiting the target RNA [32, 33]. Bioinformatics analysis revealed that miR-664b-3p could be a candidate, which contains predicted binding sites for TAF1A-AS1 and USP22. MiR-664b-3p is a tumor suppressor of HCC, which is related to the proliferation and invasion of HCC [12]. qRT-PCR results showed that knockdown of TAF1A-AS1 increased the level of miR-664b-3p in HCC cells and vice versa. And miR-664-3p could indeed influence USP22 expression in HCC cells. We further confirmed that TAF1A-AS1 interacts with miR-664b-3p by dual-luciferase reporter assay and RIP assay. Dual-luciferase reporter assay showed that miR-664b-3p was able to interact with USP22 mRNA. Finally, qRT-PCR results confirmed that TAF1A-AS1 upregulated the expression of USP22 by sponging miR-664b-3p.

Sorafenib has been identified as an inhibitor of multiple kinases and is the only effective first-line drug approved for the treatment of advanced HCC. Sorafenib achieves the purpose of killing cancer cells by inhibiting cell proliferation and anti-angiogenesis and promoting cell apoptosis [34–39]. But with the increasing use of sorafenib, tumors have developed resistance to it. The mechanisms of drug resistance are diverse. Recent studies have found that lncRNAs play an important role in the development of HCC resistance to sorafenib, and activating PI3K/mTOR can enhance the resistance of HCC to sorafenib [40–43]. mTOR plays a key role in HCC tumorigenesis and promotes the growth and progression of HCC by directly targeting the phosphorylation [44]. Our data showed that TAF1A-AS1 enhances mTOR expression and phosphorylation. In the CCK-8 and colony formation assays, the results showed that knockdown of TAF1A-AS1 enhances HCC cells sensitivity to sorafenib, and this chemosensitization effect was abolished by USP22 overexpression.

Accumulative data have supported that competing endogenous RNA (ceRNA) networks represent a type of critical mechanism regarding HCC progression and prognosis. Based on the prior reports, each ceRNA network seems to play its critical and unique role in some specific HCC biological process. For example, HOTAIR is primarily involved in cancer cell proliferation and apoptosis, while MALAT1 is largely related to cancer metastasis [45–47]. As for TAF1A-AS1, our current study supports its critical role in USP22/mTOR signaling pathway, which as a central regulator of cell growth and metabolism, critically participates in HCC resistance to sorafenib. These findings provide a novel understanding regarding chemotherapeutic failure, which would potentially facilitate further design and development of more effective regimens for HCC treatment.

Compared with traditional drug targets and proteins, gene therapy targeting lncrnas remains an emerging concept and Strategy. The current mainstream lncRNA targeting methods include small interfering RNAs (siRNAs), antisense oligonucleotides (ASOs), CRISPR/Cas9 systems and small molecule compounds [48–53]. However, most research on targeting lncRNAs is still in the preclinical stage. Based on our research results, evaluating the TAF1A-AS1 level will help screen out HCC patients resistant to sorafenib and further formulate strategies aimed at reversing sorafenib resistance. In the future, the development of targeted ASOs or small molecule compounds for TAF1A-AS1 will be a potentially effective candidate strategy for the treatment of HCC, especially in the population with high expression of TAF1A-AS1.

Our study demonstrated that TAFlA-AS1 is significantly upregulated in HCC tissues compared to adjacent normal tissues, suggesting its potential as a novel diagnosis and/or prognostic biomarker. To translate this finding into clinical practice, large-scale, multi-center prospective studies are required to establish its critical clinical thresholds. In addition, recent studies have shown that serum lncRNAs can serve as potential diagnostic and/or prognostic biomarkers for HCC [54–61]. Serum levels of lncRNAs combined with level of AFP are expected to improve the diagnosis and/or prognosis of HCC [56, 62, 63]. Specifically, future research can use readily available clinical samples such as circulating tumor cells or serum exosome to detect the expression level of TAFlA-AS1, and then improve the sensitivity, specificity, and accuracy of HCC diagnosis and/or prognosis models by integrating it with established markers such as AFP or GALAD score. Apparently, there is still a great deal of research work need to perform in the future to facilitate the translation from laboratory findings to clinical practice.

In short, TAF1A-AS1 was a tumor-promoting factor that can mediate the proliferation, metastasis and sorafenib resistance of HCC cells. Mechanistically, TAF1A-AS1 as a ceRNA competitively binds miR-664b-3p, promotes the expression of USP22 and enhances the mTOR signaling. Therefore, our findings suggest that TAF1A-AS1 could be a new therapeutic target and a biomarker for HCC.

## Conclusion

Our study confirmed that TAF1A-AS1 functions as an oncogenic lncRNA to promote proliferation, metastasis and sorafenib resistance in HCC cells. Mechanistically, we found that TAF1A-AS1 can sponge miR-664b-3p, thereby upregulating USP22 expression. In addition, TAF1A-AS1 enhances the mTOR signaling through USP22. The TAF1A-AS1-miR-664b-3p-USP22 axis is critical for the progression of HCC, and TAF1A-AS1 is expected to become a biomarker for cancer diagnosis and prognosis.

## Supplementary Information

Supplementary Material 1

Supplementary Material 2

Supplementary Material 3. Expression and transfection efficiency of TAF1A-AS1 in HCC cells. **A** The mRNA levels of TAF1A-AS1 in various liver cancer cell lines were detected by qRT-PCR. **B** The expressions of TAF1A-AS1 in SK-Hep1 or in Huh7 cells were confirmed by qRT-PCR after knockdown or overexpression of TAF1A-AS1, respectively. ***, *P* < 0.001.

Supplementary Material 4. Expression of USP22 in TCGA database and HCC clinical samples. **A** USP22 expression was analyzed in TCGA database. **B** The expression of USP22 in HCC clinical samples was determined by RT-qPCR. ***, *P* < 0.001.

## Data Availability

The original data for this article will be provided by the authors without reservation.
