# Supplementary material for: LncRNA TAF1A-AS1 regulates the progression in hepatocellular carcinoma by targeting miR-664b-3p/USP22 axis
Source: Discov Oncol. 2026 Jan 26;17:332. doi: 10.1007/s12672-026-04454-x (PMC12917072; doi:10.1007/s12672-026-04454-x)
Supplement: Supplementary file 1 — Supplementary Material 1 [file 12672_2026_4454_MOESM1_ESM.docx]

Table S2：Antibodies used in the experiments

| Antibody Name | Cat.NO. | Company |
| --- | --- | --- |
| Anti-E-cadherin | AF0131 | Affinity |
| Anti-N-cadherin | AF4039 | Affinity |
| Anti-Vimentin | AF7013 | Affinity |
| Anti-Actin | 20536 | Proteintech |
| Anti-mTOR | ab32028 | Abcam |
| Anti-p-mTOR | ab109268 | Abcam |
| Anti-USP22 | ab195289 | Abcam |
