# Supplementary material for: LncRNA TAF1A-AS1 regulates the progression in hepatocellular carcinoma by targeting miR-664b-3p/USP22 axis
Source: Discov Oncol. 2026 Jan 26;17:332. doi: 10.1007/s12672-026-04454-x (PMC12917072; doi:10.1007/s12672-026-04454-x)
Supplement: Supplementary file 2 — Supplementary Material 2 [file 12672_2026_4454_MOESM2_ESM.docx]

Table S1 Genes and primer sequences used for qRT-PCR

| Gene Name | Primers | Sequences |
| --- | --- | --- |
| *TAF1A-AS1* | forward | CTATCCTGCTGGAGACAGTGG |
|  | reverse | CCAAGATGCTGACTGTTGGT |
| *miR-664b-3p* | forward | GCCGCGTTCATTTGCCTCCCAGCCT |
| *USP22* | forward | 5ʹ-GGCGGAAGATCACCACGTAT-3ʹ |
|  | reverse | 5ʹ-TTGTTGAGACTGTCCGTGGG-3ʹ |
| *U6* | forward | 5'-CGCAAGGATGACACGCAAATTC-3' |
|  | reverse | 5'-GTGCAGGGTCCGAGGT-3' |
| *GAPDH* | forward | 5ʹ-CCACATCGCTCAGACACCAT-3ʹ |
|  | reverse | 5ʹ-ACCAGGCGCCCAATACG-3ʹ |
| *β-actin* | forward | 5'-CCTTCCTGGGCATGGAGTC-3' |
|  | reverse | 5'-TGATCTTCATTGTGCTGGGTG-3' |
