# Supplementary figures and images for: LncRNA TAF1A-AS1 regulates the progression in hepatocellular carcinoma by targeting miR-664b-3p/USP22 axis
Source: Discov Oncol. 2026 Jan 26;17:332. doi: 10.1007/s12672-026-04454-x (PMC12917072; doi:10.1007/s12672-026-04454-x)

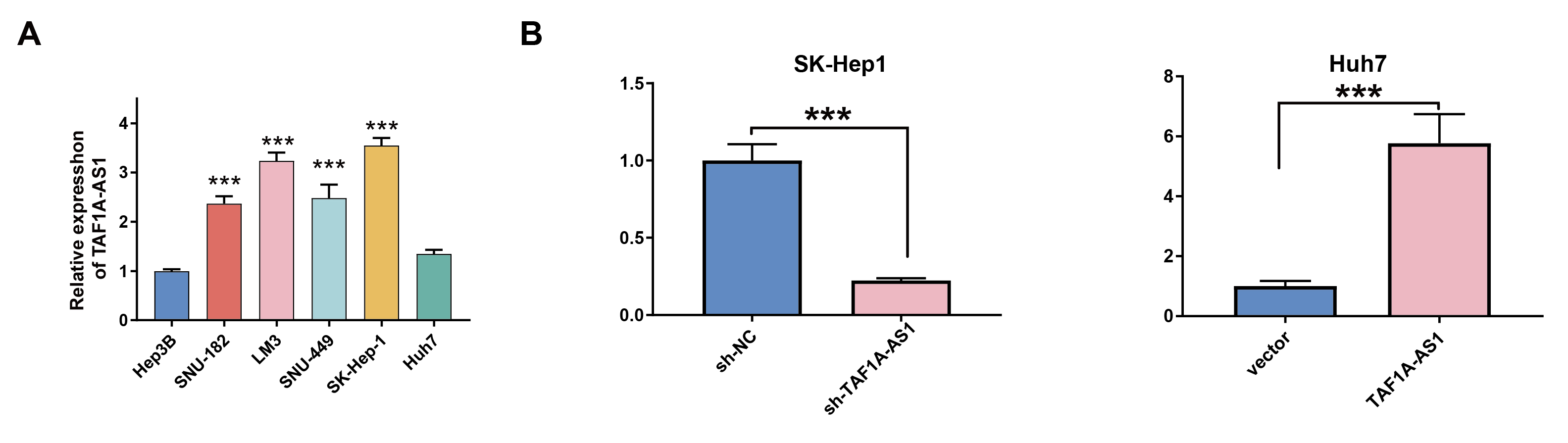

Supplement: Supplementary file 3 — Supplementary Material 3. Expression and transfection efficiency of TAF1A-AS1 in HCC cells. A The mRNA levels of TAF1A-AS1 in various liver cancer cell lines were detected by qRT-PCR. B The expressions of TAF1A-AS1 in SK-Hep1 or in Huh7 cells were confirmed by qRT-PCR after knockdown or overexpression of TAF1A-AS1, respectively. ***, P < 0.001. [file 12672_2026_4454_MOESM3_ESM.jpg]

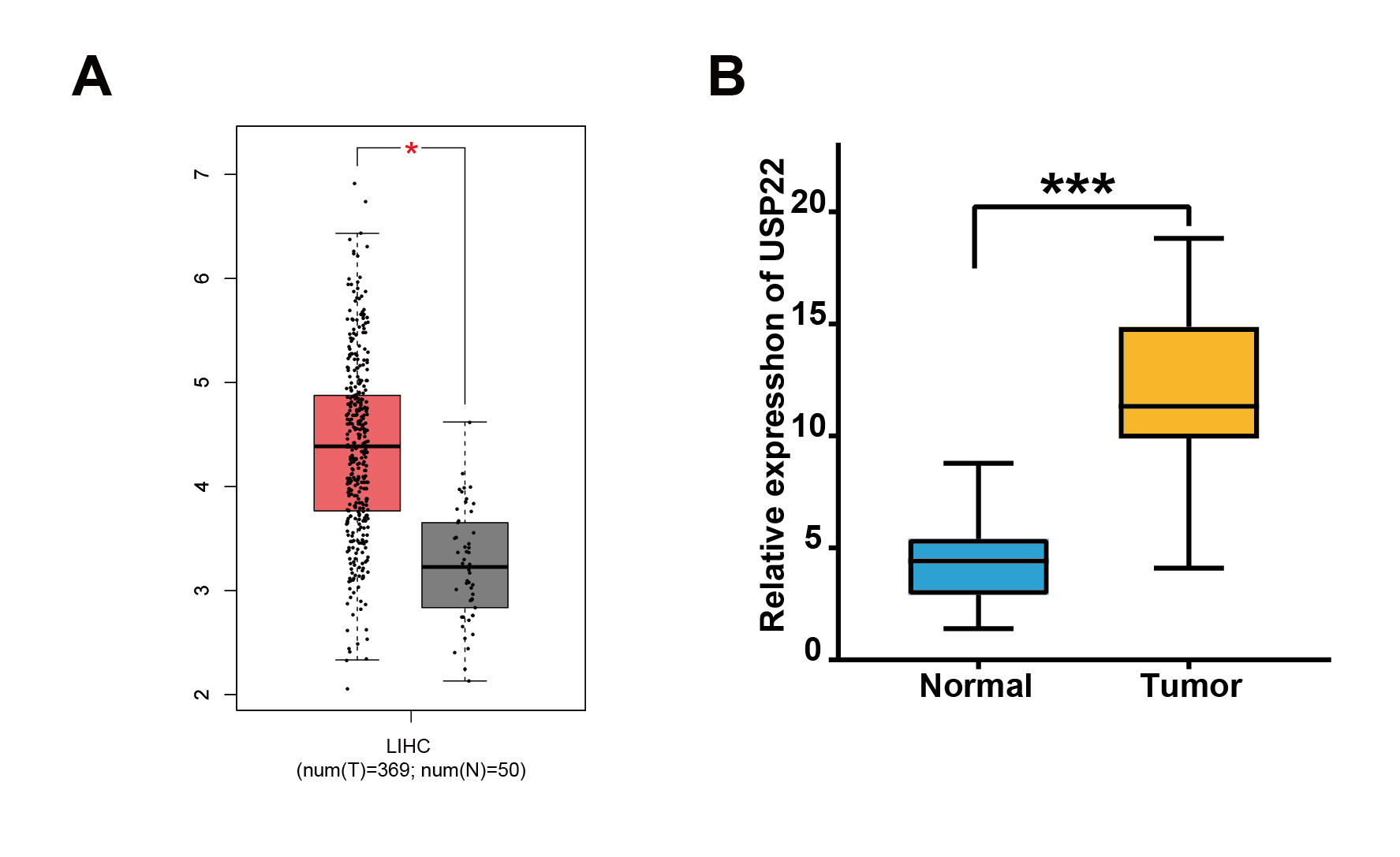

Supplement: Supplementary file 4 — Supplementary Material 4. Expression of USP22 in TCGA database and HCC clinical samples. A USP22 expression was analyzed in TCGA database. B The expression of USP22 in HCC clinical samples was determined by RT-qPCR. ***, P < 0.001. [file 12672_2026_4454_MOESM4_ESM.jpg]
